# Turn-in Flap: 10 Years’ Experience of a Single Institution in Saudi Arabia

**DOI:** 10.7759/cureus.6593

**Published:** 2020-01-07

**Authors:** Mohmmed Alkarzae, Sameer A Bafaqeeh

**Affiliations:** 1 Otolaryngology, Security Forces Hospital, Riyadh, SAU; 2 Otolaryngology Head and Neck Surgery, Facial Plastic Division, King Saud University, Riyadh, SAU

**Keywords:** rhinoplasty, plastic surgery, nose, external nasal valve, turn in flap

## Abstract

Background and objective

Cephalic excision of the lateral crus is the most used procedure in rhinoplasty when attempting to make the nasal tip smaller or narrower to improve the definition. However, due to its several drawbacks as external valve collapse, bossae formation, and alar retraction, a technique, known as the Turn-in flap, has been developed to overcome these complications and to provide better aesthetic and functional nasal tip outcomes. Therefore, we conducted this investigation to determine the long-term outcomes of such procedure.

Methods

During the period from 2007 to 2017, the charts of 120 patients who underwent the Turn-in flap procedure at King Saud University have been reviewed. The study included 42 males and 78 females with a mean age of 23 years. The follow-up duration ranged from one to 10 years with a mean duration of two years.

Results

The majority (30%) of our patients underwent Turn-in flap procedure due to combined lower lateral cartilage (LLC) convexity and bulbous tip. Satisfactory results have been observed in most cases with no post-operative complications. Only six cases required revision surgery.

Conclusions

The Turn-in folding of the cephalic part of lateral crus does not only provide functional support to the nose, but it also provides aesthetic improvement of the nasal tip with long-term satisfactory outcomes.

## Introduction

The lateral crura (LC) of the lower lateral cartilage (LLC) is an important structure of the nose both aesthetically and functionally. Aesthetically, it determines the shape, figure, volume, size, and position of the nasal tip [1]. Functionally, it shares in the structure of the external nasal valve in the anterior part of the nasal airway. This part of the alar cartilage is the cornerstone in a wide variety of maneuvers performed to correct nasal tip deformities. Accordingly, many surgical techniques aiming at reshaping the aesthetic deformities of the nasal tip are being performed mainly on the lateral crus.

Surgical excision of the cephalic portion of the lateral crura is one of the most attempted techniques for the correction of nasal tip deformities. Even though this procedure exhibits many beneficial outcomes, including reduction of crus tip volume, increase in visual gain in tip position [1-3], and narrowing of nasal tip through the medialization of the tip-defining points, there are many long-term drawbacks to it, which may consequently limit its efficacy and utility in practice. Some of these drawbacks are reduction in the size of the LC, which would weaken the alar rim and incompetence of the external nasal valve and nasal valve collapse [4-6].

In an effort to enhance the shape of nasal tip and alar arch, surgeons have attempted to preserve the integrity of the nasal cartilage through the usage of many tip-suturing procedures rather than the conventional destructive maneuvers [7-9]. That being said, the morphological variation between the lower lateral, middle, and medial crura, including concavities, convexities, irregularities, and intrinsic weakness further complicates the achievement of the optimal outcomes as regarding the nasal tip shape and the function of the external nasal valve [10-12].

Therefore, multiple surgical maneuvers have been proposed to correct the weakened or deformed lateral crura, each of which exhibits various benefits and drawbacks. These methods include simple dissection of the lateral portion of the lower lateral cartilage, suturing maneuvers of the lateral crus [13-15], reverse placement of the lateral crura, alar rim grafts [16-18], overlay or underlay graft placement [19], Turn-in flaps [2, 3, 20], or any combination of these methods.

In comparison to the cephalic excision, the Turn-in flap of the cephalic portion of the LC does not only provide aesthetic correction but also enhances the durability of the lateral crus. Moreover, the Turn-in flap procedure of the LC minimizes the tip volume, raises (increases) the tip position, and permits the medialization of the tip-defining points. Consequently, the Turn-in flap helps achieve better and more pleasant nasal tip for individuals undergoing rhinoplasty. From a functional point of view, the Turn-in flap supports the alar rim as well as treats any existing external nasal valve collapse [2, 3, 20].

Recently, many authors have been using the Turn-in flap techniques more frequently in rhinoplasty and most studies have shown promising and beneficial aesthetic and functional outcomes. However, these studies were conducted on a very limited number of patients and for a very limited amount of time. Therefore, we conducted this investigation over 10 years from 2007 to 2017 in order to determine the short- and long-term outcomes of Turn-in procedure in patients undergoing septorhinoplasty in King Abdulaziz University Hospital, Riyadh, Saudi Arabia.

## Materials and methods

This retrospective study was primarily conducted on patients undergoing open septorhinoplasty at King Abdulaziz University Hospital, King Saud University, Riyadh, Saudi Arabia during the time period from January 2007 to December 2017. All Saudi Arabian patients of 18 years of age or more, who underwent open septorhinoplasty with concave lower lateral crura, convex lower lateral crura, weak or thin lower lateral crura, or external valve collapse, were considered eligible for participation in our study.

All eligible participants who were willing to participate in our study were enrolled. Non-Saudi Arabians and patients below the age of 18 years of age or who underwent rhinoplasty previously were not included. Written informed consent was taken from each study participant prior to conducting the study.

**Figure 1:**
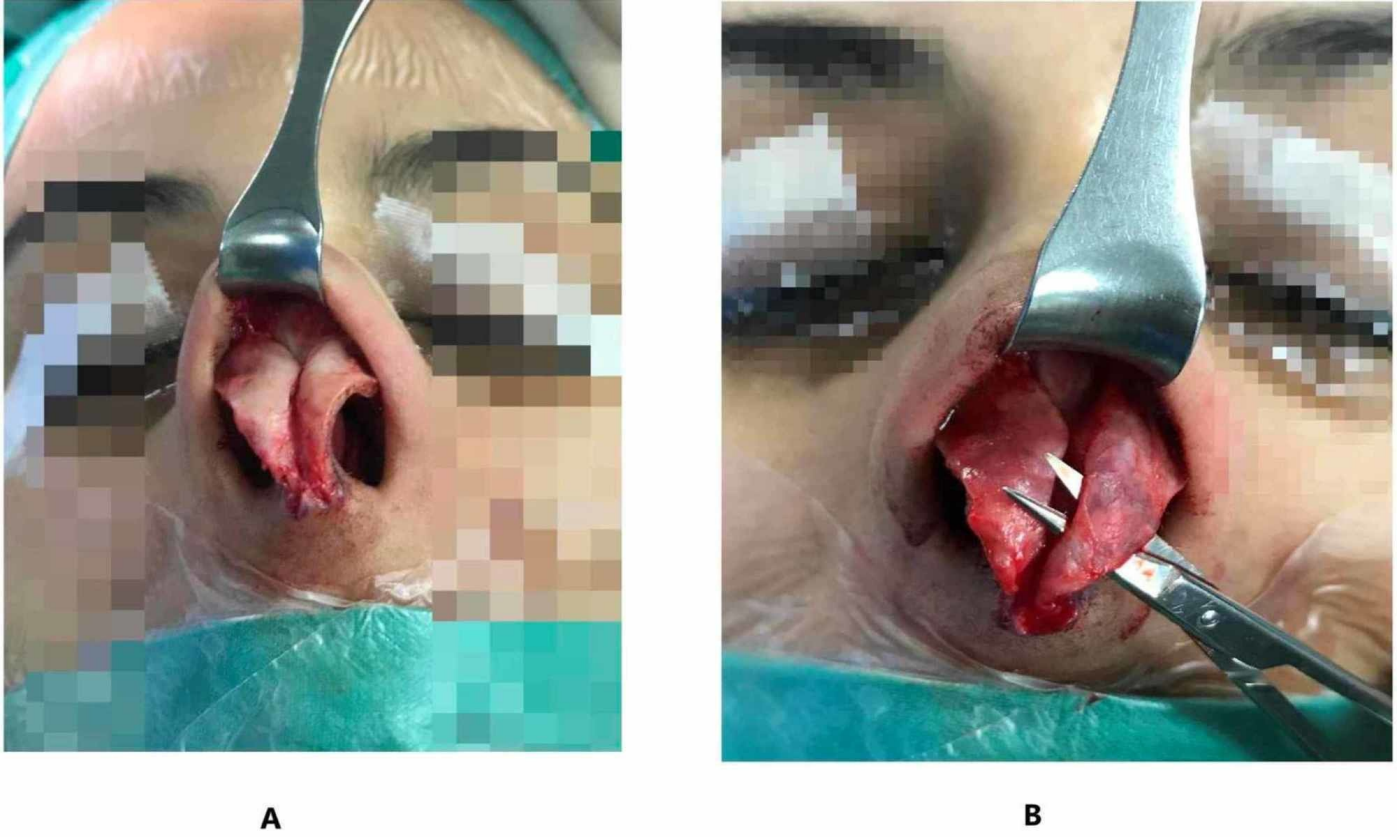
Intraoperative surgical steps (A) Full visualization of lower lateral crura. (B) Dissection of the mucosa underlying lower lateral crura.

We used the Turn-in flap procedure in all patients who were undergoing septorhinoplasty (see Figure 1). Procedure was carried out under general anesthesia. An Inverted-V incision and marginal incisions were used, where to elevate the columellar flaps, sharp curved scissors were used. Meanwhile, in order to minimize the risk of folding over of the vestibular skin, the dissection of the nasal mucosa was carried out from the inner side of the lateral crura. At this point in the procedure, the authors were able to evaluate the shape, width, thickness, orientation, and symmetry of the lateral crura, accurately.

**Figure 2:**
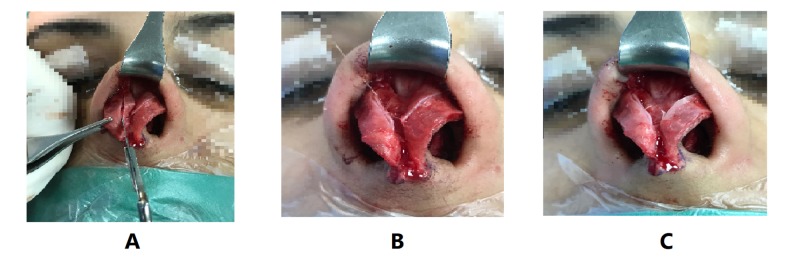
Intraoperative surgical steps (A) A complete incision of the cephalic portion of the lower lateral crura. (B) The free cephalic portion of the lateral crus was turned in and folded against its pivot points. (C) Three mattress sutures using 5-0 PDS.

During the Turn-in flap procedure, all the soft tissues surrounding medial crura, lateral crura and the domes were dissected in order to enhance the visualization of the whole lateral crus (see Figure 2). Meanwhile, the complete incision of the cephalic portion of the lateral crus was made following a straight line using a No. 15 blade, where a minimum of 7-8 mm of the cartilage was preserved. The free cephalic portion of the lateral crus was turned in and folded against its pivot points. Three mattress sutures using 5-0 PDS were enough to fixate the folded cephalic portion of the lateral crura. The first suture was in the midline, the second was close to the dome, and the third was close to the lateral margin. Upon tying the sutures, both segments of the lateral crus were joined in a ‘sandwich’ fashion. An objective assessment of the outcomes of the procedure was carried out for all patients, postoperatively.

Statistical Package for Social Sciences (SPSS) program version 23.0 (IBM Corp., Armonk, NY) was used for statistical analysis. A descriptive statistical data was presented by mean values, frequency distributions and percentages.

## Results

A total of 120 patients who fulfilled the inclusion criteria were included in our analysis. A total of 42 (35%) patients were males and 78 (65%) were females. The mean age of the included participants was 23 years. The follow-up duration of our study ranged from one year to 10 years with an average duration of two years.

Our sample was divided into 14 groups according to the preoperative examination and indications for Turn-in flap technique (Table 1). Most of our patients underwent Turn-in flap technique due to combined LLC convexity and bulbous tip (30%) followed by combined LLC concavity and weak cartilage (13.3%), and bulbous tip (10%). On the other hand, only one patient presented with external nasal valve collapse, LLC concavity, weak cartilage, and bulbous tip (0.83%).

**Table 1:** Stratification of the study population according to the presentation and indication for the Turn-in folding technique LLC: Lower lateral cartilage

Groups	Frequency (%)	Required Revision (%)
LLC Convexity + bulbous tip	36 (30)	1 (2.8)
LLC Concavity + weak cartilage	16 (13.3)	-
Bulbous tip	12 (10.0)	2 (16.7)
External valve collapse + LLC concavity + weak cartilage	12 (10.0)	1 (8.3)
Weak cartilage	10 (8.3)	-
External valve collapse + weak cartilage	9 (7.5)	1 (11.1)
No pre-operative deformity	7 (5.8)	-
External valve collapse + LLC convexity + weak cartilage + bulbous tip	5 (4.2)	-
External valve collapse + LLC convex + bulbous tip	5 (4.2)	-
LLC Convexity + weak cartilage + bulbous tip	4 (3.3)	1 (25.0)
External valve collapse + bulbous tip	1 (0.83)	-
LLC Concavity + weak cartilage + bulbous tip	1 (0.83)	-
LLC Convexity	1 (0.83)	-
External valve collapse + LLC concavity + weak cartilage + bulbous tip	1 (0.83)	-
Total	120	6

The Turn-in flap technique resulted in a symmetrical reduction of the LLC. There was no evident additional bulk in the lateral sidewall and narrowing of the nasal vestibule was not an issue. In terms of postoperative outcomes, the procedure was successful with no post-operative infection, suture reaction or visualization, or alar collapse. However, only six cases (5%) required revision surgery. The group with the highest frequency for revision was the bulbous tip group (16.7%) (see Table 1).

## Discussion

Even though cephalic excision of the lateral crus is the most commonly used procedure in septorhinoplasty to improve nasal tip deformities, it may result in weakness of the lateral crus with subsequent external nasal valve dysfunction. That being said, in certain circumstances when a substantial part of the cephalic portion of the LLC has been excised, severe external nasal valve collapse, bossae formation, alar retraction, and alar collapse may occur [21-23].

In an attempt to overcome the aforementioned complications and in order to provide the surgeon with a wide margin of safety, the Turn-in flap procedure was first proposed by Tellioglu and Cimen where the authors found that cephalic narrowing of the LLC can result in better nasal tip and nasal base support as well as a strength [3]. This procedure can be performed upon patients with weak LLC and wide lateral crus.

The Turn-in flap is a non-destructive, incremental, and reversible procedure carried out during rhinoplasty and it has been proven effective in correcting excessively convex and concave LLC with horizontal mattress sutures with no need for further grafting material [2, 3, 24].

Tellioglu and Cimen were the first to describe this procedure in 2007 where the turn-in folding of the cephalic part of the LLC was used to support the alar rim on 32 patients with satisfactory outcomes in all cases. Afterward, Murakami et al. reported similar technique in 2009 on 18 patients to strengthen the alar cartilage after cephalic trimming [2, 3]. In 2010, Sazgar used the same technique during the management of 23 cases of the drooping nose during open rhinoplasty [24]. In 2012, Apaydin modified the Turn-in flap procedure to reshape and add structure to the LLC to suspend the internal nasal valve in 24 patients [20].

Herein, we investigated the use of the Turn-in flap technique in a total of 120 patients during the period from 2007 to 2017 at the King Saud University Hospital. To the best of our knowledge, this is the largest study ever conducted to investigate the postoperative outcomes of patients undergoing Turn-in flap procedure during septorhinoplasty.

Our population was made up of 35% males and 65% of females with a mean age of 23 years. This is comparable to the previous reports [2, 3, 20, 24]. Our population was followed up for a mean duration of two years (one to 10 years). This is by far the first study to investigate the postoperative outcomes of the Turn-in flap technique over a long-term scale. The previous investigations of Apaydin, Tellioglu and Cimen, Sazgar, and Murakami et al. reported a mean follow-up time of 14, 12, 11, and nine months, respectively [2, 3, 20, 24].

In comparison to the previous reports, the main reason for performing the Turn-in flap procedure in our population was due to the presentation with combined LLC convexity and bulbous tip (30%). On the other hand, only one patient presented with external nasal valve collapse, lower lateral cartilage concavity, weak cartilage, and bulbous tip, which underwent the Turn-in flap procedure with satisfactory outcomes and no evident complications.

The procedure was carried out successfully in all our cases, where we noted a symmetric reduction of the lower lateral cartilage. This goes in line with the aforementioned reports, where successful and satisfactory results had been observed in the majority of cases [2, 3, 20, 24].

In terms of post-operative assessment, none of our patients presented with evident additional bulk in the lateral sidewalls or narrowing of the nasal vestibule. Moreover, none of our population had post-operative infections, suture reaction, or alar collapse. Only six cases (5%) required revision surgery. These cases came to our hospital with multiple presentations: one case presented with LLC convexity and bulbous nasal tip; one case presented with LLC concavity and weak cartilage; one case presented with external nasal valve collapse and weak cartilage; one case presented with LLC convexity, weak cartilage, and bulbous tip; and two cases presented with bulbous tip.

## Conclusions

In conclusion, the Turn-in flap was noted to provide an improvement over the classic cephalic excision of the lateral crus in tip refinement surgeries as well as in reinforcing the external nasal valve. Turn-in flap is a reliable technique especially in convex lower lateral cartilage combined with bulbous tip. This goes in line with the literature. Meanwhile, the major drawback to this procedure is the inapplicability in those who have undergone prior over-resection of the lateral crus due to the unavailability of enough cartilage to be used for the turn-in flap.
